# Autoimmune markers in screening for orbital inflammatory disease

**DOI:** 10.1038/s41433-022-02068-5

**Published:** 2022-04-19

**Authors:** Terence Ang, Valerie Juniat, Dinesh Selva

**Affiliations:** 1 Discipline of Ophthalmology and Visual Sciences, The University of Adelaide, Adelaide, SA Australia; 2 Department of Ophthalmology, The Royal Adelaide Hospital, Adelaide, SA Australia

**Keywords:** Biomarkers, Autoimmune diseases, Eye diseases

## Abstract

**Purpose:**

Immunogenic causes of inflammation may be difficult to differentiate in the work-up of orbital inflammatory disease. The study aims to investigate the utility of autoimmune markers in the screening for orbital inflammation. Markers studied included angiotensin-converting enzyme (ACE), antinuclear antibody (ANA), anti-neutrophilic cytoplasmic autoantibodies (ANCA), extractable nuclear antigen (ENA), anti-cyclic citrullinated peptide (Anti-CCP) and anti-double stranded DNA antibody (Anti-dsDNA antibody).

**Methods:**

A retrospective single-centre study of consecutive patients with non-infective orbital inflammation screened for autoimmune markers at presentation. Serology was interpreted alongside clinical course and other investigations (e.g. radiographic features and histopathology). Tabulated data and Pearson’s Chi-square allowed analysis of trends between serology, diagnosis and the decision to biopsy.

**Results:**

79 patients, between 1999 and 2021, were included (50 females, mean age was 50.4 ± 17.4 years). 28 (34.6%) patients had specific orbital inflammation and 53 (65.4%) patients had non-specific orbital inflammation (NSOI). Of the 12 patients with positive serology and a specific diagnosis, only 5 (41.7%) patients had concordant serological results. There was no association between serology results and the patient undergoing biopsy (*P* = 0.651). Serology was unable to exclude nor differentiate NSOI from other specific conditions and ANA had limited discriminatory value between specific conditions and NSOI.

**Conclusion:**

Serological testing alone may not provide a clear direction for further investigation of orbital inflammation and a biopsy may occur independently of the serological results. The value of autoimmune markers may lie in subsequent follow-up as patients may develop suggestive symptoms after an indeterminate positive result or initially seronegative disease.

## Introduction

Orbital inflammatory disease (OID) may be due to specific localised or systemic inflammatory diseases, such as sarcoidosis, granulomatosis with polyangiitis (GPA) and IgG4-related orbital disease (IgG4-ROD) [1–4]. If no identifiable underlying cause can be determined, a diagnosis of non-specific orbital inflammation (NSOI) can be made [5]. Serology can be used to help diagnose various immunogenic causes of orbital inflammation. However, in situations of diagnostic uncertainty, histopathological analysis may still be required to confirm or support the diagnosis [2, 3].

The aim of this study was to investigate the utility of various autoimmune markers in screening for immunogenic OID. Autoimmune markers studied included angiotensin-converting enzyme (ACE), antinuclear antibody (ANA), anti-neutrophilic cytoplasmic autoantibodies (ANCA), extractable nuclear antigen (ENA), anti-cyclic citrullinated peptide (Anti-CCP) and anti-double stranded DNA antibody (Anti-dsDNA antibody).

## Methods

This was a single-centre cross-sectional retrospective study of patients with orbital inflammation. Inclusion criteria were patients >18 years of age with clinical, radiological and/or histologically confirmed OID, with autoimmune markers conducted at initial presentation. Exclusion criteria included patients without any autoimmune markers screened; patients with thyroid eye disease; and patients diagnosed with infectious orbital disease (e.g.: orbital cellulitis). Patients with infectious orbital disease were excluded based on suggestive clinico-radiological findings and response to systemic antibiotics. Patients included in this study were categorised as having a specific diagnosis (e.g.: sarcoidosis, orbital GPA) if there was clinico-radiological and/or histological evidence of a specific diagnosis. For example, a diagnosis of IgG4-ROD was assessed according to the Comprehensive diagnostic (CD) criteria for IgG4-related disease; and GPA was diagnosed as according to the 1990 American College of Rheumatology (ACR) criteria [6, 7]. Meanwhile, patients without any identifiable local or systemic cause were deemed NSOI (i.e. a diagnosis of exclusion). A diagnosis of NSOI was further evidenced by supporting clinico-radiological (including lack of radiologically-specific changes seen in specific diagnoses, such as sino-nasal involvement in GPA) and/or histopathological presentation and response to corticosteroid therapy [8].

Patients were identified from the Oculoplastics Unit at the Royal Adelaide Hospital (Adelaide, Australia). Data were sourced from paper and electronic records. Data collected included patient demographics (age at presentation, gender, past medical history and relevant medications), diagnostic details (diagnosis and orbital structure involved) and autoimmune markers (ACE, ANA, ENA, ANCA, anti-dsDNA antibody and anti-CCP). Patients were classified as having positive or negative serology. Autoimmune markers were deemed positive if a positive marker or titre was reported at presentation. At our institution, ANA was reported positive at titres above 1/40. Patients were further categorised according to their specific or non-specific diagnosis (i.e. NSOI) and those with a biopsy were also noted.

Within the typical diagnostic work-up of OID, laboratory investigations included a combination of a complete blood count, erythrocyte sedimentation rate, C-reactive protein, thyroid function tests (including anti-thyroid antibodies) and any indicated autoimmune serology. Radiological and/or histological findings provided further diagnostic information [9, 10].

Statistical analysis was performed with SPSS (IBM corporation, New York). Where applicable, results are expressed as mean ± standard deviation (σ) and presented in relevant tables. Pearson’s chi-square test allowed analysis between the incidence of autoimmune marker results and the incidence of patients undergoing a biopsy (*P* < 0.05 deemed statistically significant). All research was conducted in accordance with the Declaration of Helsinki and was approved by our Institutional Review Board with a waiver of consent granted.

## Results

### Demographics

79 patients, between 1999 and 2021, were analysed in this study. 50 (63.3%) were females, 29 (36.7%) were males and the mean age was 50.4 ± 17.4 years (range: 18–86 years). 28 (35.4%) patients had a specific diagnosis. The remaining 51 (64.6%) patients had NSOI, with 20 (39.2%) classified as diffuse inflammation, 20 (39.2%) as dacryoadenitis and 11 (21.6%) as myositis. A summary of diagnoses is provided in Table 1. Mean age was similar between both groups (NSOI: 49.5 ± 17.1 years; specific inflammation: 52.1 ± 18.2 years). Of the 47 (59.3%) patients with a biopsy, 16 (34.0%) patients had specific OID and 31 (66.0%) patients had NSOI. Mean follow-up period was 18.5 ± 32.7 months (range: 1 week–192 months) for NSOI patients and 18.6 ± 20.6 months (range: 3 days–75 months) for specific conditions.

**Table 1:** Summary of diagnoses.

Diagnosis		Number of patients
NSOI	Diffuse	20
	Dacryoadenitis	20
	Myositis	11
Specific inflammatory conditions	RLH	6
	GPA	6
	IgG4-ROD	5
	Drug-induced^a^	3
	Sarcoidosis	3
	Sjogren’s syndrome	2
	EGPA	1
	Overlap syndrome	1
	SLE	1

*NSOI* non-specific orbital inflammation, *GPA* granulomatosis with polyangiitis, *EGPA* Eosinophilic granulomatosis with polyangiitis, *RLH* reactive lymphoid hyperplasia, *IgG4-ROD* IgG4-related orbital disease, *SLE* Systemic lupus erythematosus.

^a^Drugs implicated include statins, bisphosphonate and hyalase allergy.

### Association between serology and diagnosis

**Fig. 1:**
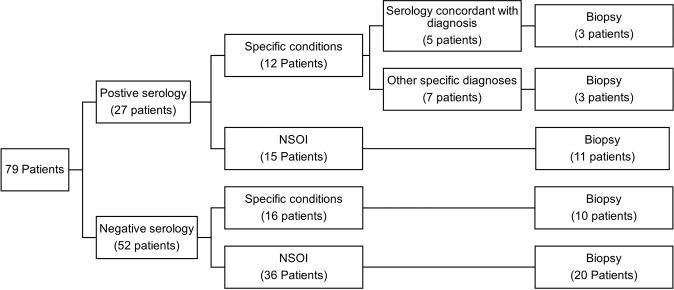
Utility of autoimmune markers according to serological results. Flowchart demonstrating categorisation of patients according to serological results and subsequent diagnoses. NSOI non-specific orbital inflammation.

Figure 1 demonstrates the distribution of the 79 patients screened with autoimmune markers at presentation.

Biopsies were performed in patients where clinical and radiological findings were inconclusive, in patients with positive serology in the absence of known systemic involvement, and in patients with treatment-resistant disease. Overall, of the 27 patients with a positive serology, 17 (63.0%) underwent a biopsy. Meanwhile, of the 52 patients with negative serology, 30 (57.7%) patients underwent a biopsy. Chi-square analysis demonstrated no significant association between serology result (positive or negative) and the patient undergoing a biopsy (*P* = 0.651).

Of the 12 patients with positive serology and diagnosed with a specific condition, only 5 (41.7%) of these patients had serology supporting the final diagnosis. These included 2 (40%) patients with first onset sarcoidosis (no previously known systemic involvement), 1 (20%) patient later diagnosed with Sjogren’s syndrome (SS), 1 (20%) patient diagnosed with overlap syndrome at follow-up and 1 (20%) patient demonstrating ANA positivity in SLE-associated bilateral dacryoadenitis. Of these 5 patients with concordant serology, 3 (60%) proceeded to a biopsy, including the 2 patients with sarcoidosis and 1 patient with overlap syndrome.

Of the remaining 7 patients with positive serology and a specific diagnosis, 3 (42.9%) patients proceeded to a biopsy, including 2 patients with IgG4-ROD and 1 patient with RLH. The remaining 4 (57.1%) patients had a clinico-radiological diagnosis for their specific condition (e.g.: onset of myositis symptoms shortly after administration of bisphosphonates).

### Analysis of individual markers

A summary of individual autoimmune marker findings is reported in Table 2. Only 2 patients had elevated ACE levels, representing 66.6% of those with sarcoidosis. 73 patients had ANA screening and 19 (26.0%) patients were positive. Of those with ANA positivity, 13 (68.4%) patients had NSOI, representing 26% of the 50 NSOI patients with a screening ANA. Within this NSOI group, there were no patients with a history of autoimmune connective tissue disease.

**Table 2:** Summary of diagnoses and autoimmune results.

Diagnosis	Positive result/patients with respective diagnosis tested (%)					
	ACE	ANA	ANCA	ENA	Anti-CCP	Anti-dsDNA
NSOI	0/47 (0)	13/50 (26)	4/49 (8.2)	0/34 (0)	0/16 (0)	0/23 (0)
RLH	0/4 (0)	0/5 (0)	0/4 (0)	0/5 (0)	0/2 (0)	0/4 (0)
GPA	0/1 (0)	1/3 (33.3)	0/6 (0)	0/1 (0)	0/1 (0)	0/2 (0)
Drug-induced	0/2 (0)	2/3 (66.6)	0/2 (0)	1/3 (33.3)	0/1 (0)	0/3 (0)
IgG4-ROD	0/4 (0)	2/5 (40)	2/3 (66.6)	1/3 (33.3)	0/1 (0)	0/2 (0)
Sarcoidosis	2/3 (66.6)	0/3 (0)	0/3 (0)	0/2 (0)	0/2 (0)	0/1 (0)
Sjogren’s Syndrome	0/1 (0)	0/2 (0)	0/2 (0)	0/2 (0)	1/1 (100)	0/1 (0)
EGPA	0/1 (0)	–	0/1 (0)	0/1 (0)	–	–
Overlap Syndrome	0/1 (0)	0/1 (0)	0/1 (0)	1/1 (100)	0/1 (0)	0/1 (0)
SLE	0/1 (0)	1/1 (100)	0/1 (0)	0/1 (0)	0/1 (0)	0/1 (0)
Total patients	65	73	72	53	26	38

*NSOI* non-specific orbital inflammation, *ACE* Angiotensin-converting enzyme, *ANA* anti-nuclear antibody, *ENA* Extractable Nuclear Antigen, *ANCA* anti-neutrophilic cytoplasmic antibody, *Anti-dsDNA* Anti-double stranded DNA antibody, *Anti-CCP* Anti-cyclic citrullinated peptide.

72 patients were tested for ANCA and 6 (8.3%) reported ANCA positivity. These 6 patients, including 4 patients which were diagnosed with NSOI, had weak ANCA positivity, and ultimately had a negative myeloperoxidase and proteinase 3 (PR3). All 6 patients with orbital GPA reported negative ANCA titres at presentation. However, 5 patients had previously known systemic disease and had decreasing or negative titres on maintenance therapy. 1 patient presented with new-onset sino-orbital disease, but seroconversion only occurred at 3 months follow-up. Negative ANCA was observed in the single patient with eosinophilic granulomatosis with polyangiitis (EGPA).

ENA was screened in 53 patients and was positive in 3 (5.7%) patients. Of these 3 patients, 1 (33.3%) patient with positive anti-Ku antibody developed systemic symptoms consistent with overlap syndrome. Both patients with SS had negative anti-Ro or anti-La antibodies. Anti-CCP was screened in 26 patients with 1 (3.8%) positive result, which was observed in a case of SS. This patient was subsequently diagnosed with rheumatoid arthritis and secondary SS.

Finally, none of the 38 patients tested for anti-dsDNA had positive results.

### Association between serology and orbital structure

**Fig. 2:**
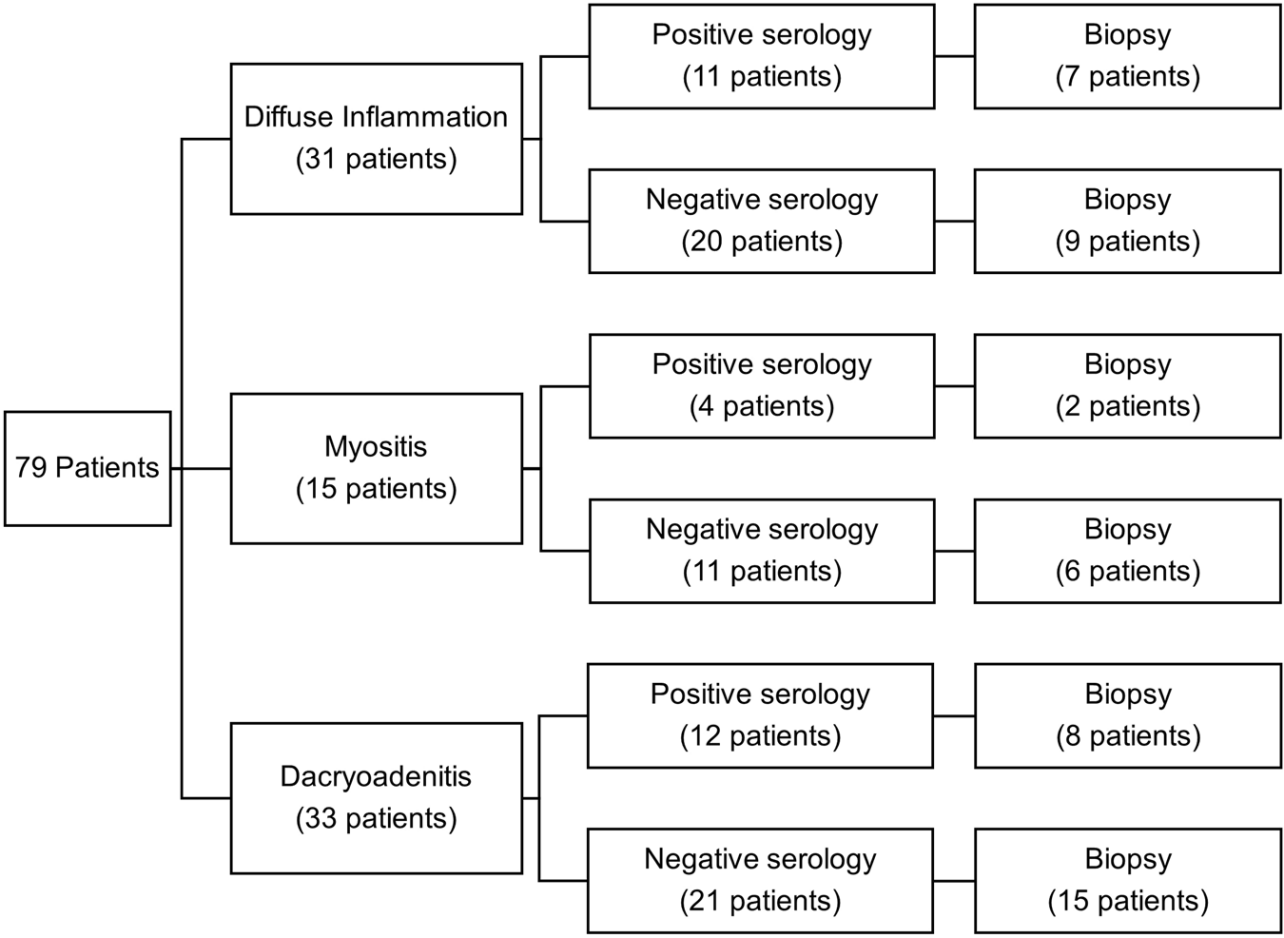
Utility of autoimmune markers according to affected orbital structures. Flowchart demonstrating categorisation according to orbital structure and serological results.

Figure 2 depicts the utility of autoimmune markers according to affected orbital structure. Within this study, 31 (39.2%) patients had diffuse inflammation, 15 (19.0%) had myositis and 33 (41.8%) had dacryoadenitis. 16 (51.6%), 8 (53.3%) and 23 (69.7%) patients within these groups, respectively, had a biopsy. Chi-square analysis demonstrated statistically insignificant associations between serology result and the patient undergoing a biopsy for diffuse inflammation (*P* = 0.320), myositis (*P* = 0.876) and dacryoadenitis (*P* = 0.775).

## Discussion

The results show that only a small proportion of patients with specific immunogenic OID are supported by concordant serological results at time of presentation. The results of serological testing do not specify a diagnosis. They are an adjunctive investigation, used along with clinical findings and radiological tests, for diagnosing specific OID (e.g.: GPA is not solely diagnosed by ANCA positivity, nor is sarcoidosis diagnosed solely by elevated ACE levels). In addition, many of these patients may have previously known systemic involvement; have a diagnosis only confirmed at follow-up; or may still proceed to a biopsy for histopathological diagnosis. Serological testing was unable to exclude nor differentiate NSOI from other specific causes of orbital inflammation. Even after differentiation of the subtype of orbital inflammation (diffuse, myositis or dacryoadenitis), there was no association between serology and the patient undergoing a biopsy. Thus, serology alone was unlikely to influence the decision to undergo a biopsy, enforcing the point that the decision to biopsy remains multifactorial. For example, biopsy may be indicated in treatment-resistant disease, or less likely to be conducted in situations where a clinico-radiological diagnosis may be made (e.g.: a previous diagnosis of a systemic disease).

Regarding the individual markers, elevated ACE levels supported sarcoidosis, but normal levels did not exclude disease. ANA was a highly non-specific marker with limited utility. ANCA screening has limited value in patients with known systemic GPA on maintenance therapy, and seroconversion for first presentation may only be revealed at future follow-up. In patients with specific markers, such as anti-CCP or anti-Ku antibody, follow-up may reveal systemic manifestations confirming a diagnosis.

Within the literature, the limitations and diagnostic complexity when approaching suspected cases of immunogenic OID have been outlined [11, 12]. Mombaerts et al. has previously reported that serological studies have limited diagnostic value in orbital inflammation as results may be non-specific, disease is generally localised to the orbit and severe disease would be required to create a positive assay. Thus, as evidenced by our findings and the literature, positive markers are considered only as corroborative evidence [3]. This study further highlights the limitations of serological testing and suggests that in many circumstances such results do not strongly influence the diagnostic pathway and decision to proceed to a biopsy.

Choice of autoimmune markers in the diagnostic approach of orbital inflammation should be guided by clinico-radiological presentation [2]. Elevated ACE levels reportedly occurs in 60–90% of active sarcoidosis, with levels generally proportional to severity [13–15]. Thus, normal ACE levels may not distinguish between less active and absent disease [14]. Furthermore, systemic disease may subsequently be revealed in 9–50% of patients, but ACE levels may remain normal [13, 16]. ACE may also be increased in other conditions, such as orbital lymphoma and NSOI [17, 18]. Elevated ACE levels are reportedly a predictor of positive yield on biopsy [19]. Tissue biopsy should be obtained in patients with supportive clinico-radiological features and elevated serum ACE, as was evident in our cases [11].

Within our study, ANA had limited discriminatory value between specific and non-specific disease [20]. Although ANA positivity is reported in 59–85% of SS patients, both SS patients in our study had negative ANA, albeit in a small represented sample [21]. ANA is a poor screening test for primary SS, as 23% have ANA negativity and 9% have a negative ANA and ENA [22]. Positive ANA may also occur in other autoimmune and non-rheumatic diseases, and are detectable in up to 20–30% of healthy adults, with significantly elevated levels observed in 2.5% of the general population [23–25]. Although, patients may exhibit positive ANA years before a diagnosis of autoimmune disease, studies have reported that many ANA-positive patients do not have autoimmune disease nor do they have an increased likelihood of future autoimmune disease [3, 24]. Furthermore, although thyroid eye disease was excluded from this study, interpretation of ANA should also consider autoimmune thyroid disease, as ANA positivity may also occur in these cases [26, 27]. Thus, thyroid function tests should be considered alongside any indicated autoimmune markers.

Positive cANCA may be observed in 90% of active generalised GPA [28–32]. However, ANCA testing is less sensitive in localised and isolated sino-orbital GPA, and titres can become negative after treatment, as was evident in our study [11, 32–40]. Patients with new-onset disease may present with negative ANCA, but may seroconvert at follow-up [39, 41, 42]. It has been estimated that only 32–35% of patients with known sino-orbital disease have cANCA-positivity at presentation, with an additional 10% converting 6–24 months after initial presentation [39, 41]. Although biopsy can help confirm the diagnosis, histopathology may reveal granulomatous inflammation without vasculitis, a non-specific finding which may also be indicative of sarcoidosis, NSOI or aspergillosis [33, 43, 44].

ENAs encompass a range of specific autoantibodies used to help diagnose autoimmune connective tissue diseases [45]. For example, anti-Ro antibodies are found in 50–75% and 15% of patients with primary and secondary SS, respectively [46–48]. However, positive anti-Ro antibodies may be observed in a range of other autoimmune disorders and in health individuals [46, 47]. Within our study, a patient with positive anti-Ku antibody developed symptoms suggestive of overlap syndrome.

Anti-CCP is typically a specific marker for rheumatoid arthritis. In addition, high anti-CCP levels are also observed in the minority of patients with primary SS [49–51]. Only 1 patient with SS had a positive anti-CCP, and follow-up revealed early rheumatoid arthritis. Anti-CCP in patients with arthritis may help predict progression to rheumatoid arthritis [52]. Anti-dsDNA antibody is specific for SLE, which remains a cause of orbital inflammation [53, 54]. However, unless there are clinical features supporting a high pre-test probability of SLE, anti-dsDNA antibody is not a recommended investigation in situations of ANA negativity [55].

The main limitation of this study is the small sample sizes represented for each specific inflammatory condition. Secondly, patients diagnosed with GPA were on maintenance therapy which are known to reduce or cause negative ANCA titres. Finally, although the heterogeneity of autoimmune markers tested at presentation and subsequent follow-up reflect the clinical pre-test probability of a specific diagnosis, various orbital conditions, such as limited GPA-related orbital disease, are known to present with seronegative disease [56]. There is limited literature regarding the characteristics of seroconversion of autoimmune markers in patients with orbital disease. Thus, future studies may involve longitudinal analysis of patients screened with autoimmune markers to investigate the utility of serial testing in initially seronegative disease.

In conclusion, autoimmune markers often have limited utility in screening for immunogenic OID. The choice of autoimmune markers in the initial diagnostic work-up of OID should be selective and rationalised by the clinico-radiological presentation, as a full panel approach may yield non-specific findings. In many circumstances, the decision to proceed with a biopsy occurs independently of the serological result. The value of autoimmune markers may lie in subsequent follow-up as patients may later develop suggestive clinical features after a non-specific positive result or in initially seronegative disease.

## Summary

### What was known before

- Immunogenic causes of orbital inflammation may be difficult to differentiate.
- Autoimmune markers are commonly used to screen for causes of orbital inflammation.
- In situations of diagnostic uncertainty, histopathological analysis may still be required to support or confirm the diagnosis.

### What this study adds

- Only a small proportion of patients with a specific diagnosis may have positive concordant serological results on initial assessment.
- Serological testing alone may not provide a clear direction for further investigation of orbital inflammation.
- The value of serological testing may lie in subsequent follow-up to detect initially indeterminate positive findings or seronegative disease.

## Data Availability

The datasets generated during and/or analysed during the current study are not publicly available due to containing information that could compromise patient privacy but are available from the corresponding author on reasonable request.
